# Draft genome sequence of *Pseudomonas aeruginosa* SAU_MI_1F1 isolated from feces of cattle in Dhaka, Bangladesh

**DOI:** 10.1128/mra.00266-26

**Published:** 2026-06-15

**Authors:** Arpita Biswas, Md Hafizur Rahman, Md. Mominul Islam, Alich Mondal, Rahnuma Tabassum Taqwa, Fatema Tuj Zohura, Md. Rashedul Islam, Mahfuzul Islam

**Affiliations:** 1 Department of Microbiology and Parasitology, Sher-e-Bangla Agricultural University 54494, Dhaka, Bangladesh; 2 AMR Reference Laboratory (Research), Animal Health Research Division, Bangladesh Livestock Research Institute 560480, Dhaka, Bangladesh; 3 Department of Pathology, Sher-e-Bangla Agricultural University 54494, Dhaka, Bangladesh; 4 Department of Surgery and Theriogenology, Sher-e-Bangla Agricultural University 54494, Dhaka, Bangladesh; University of Maryland School of Medicine, Baltimore, Maryland, USA

**Keywords:** *Pseudomonas aeruginosa*, cattle, feces, antibiotic resistance genes

## Abstract

We report a draft genome sequence of *Pseudomonas aeruginosa* isolated from feces of cattle in Dhaka, Bangladesh. The 6.4 Mb genome, sequenced using Illumina NextSeq, contains 5,843 coding sequences, including putative antibiotic resistance genes.

## ANNOUNCEMENT

*Pseudomonas aeruginosa* is a highly adaptable opportunistic pathogen characterized by remarkable genetic diversity, enabling its persistence across a wide range of ecological niches, including healthcare environments as well as natural reservoirs, such as soil, water, and animal hosts (1). It is a major cause of infections among immunocompromised individuals and is well known for its extensive intrinsic and acquired resistance to multiple antibiotics (2).

The draft genome sequence of *Pseudomonas aeruginosa* isolated from an apparently healthy feedlot dairy cow in Dhaka (23° 37′ to 23° 47′ north latitudes and 90° 13′ to 90° 29′ east longitudes), Bangladesh, is reported here. Freshly voided bovine fecal samples were aseptically collected using sterile cotton swabs and immediately transferred into 9 mL of peptone water for enrichment, followed by incubation at 37°C for 6 h under aerobic conditions. The enriched culture was subsequently streaked onto a nutrient agar plate and incubated at 37°C for 20 h. A single, well-isolated colony without visible contamination was selected for downstream analysis. Species identification was confirmed by MALDI-TOF mass spectrometry. Genomic DNA was extracted from an overnight nutrient broth culture incubated at 37°C using the QIAamp DNA Mini Kit (QIAGEN, Germany).

The genome was sequenced on an Illumina NextSeq 2000 platform using the Nextera DNA Flex Library Prep Kit (Illumina, San Diego, CA, USA) with a paired-end sequencing (2 × 150 bp) approach that generated 5,222,578 paired-end reads. The library preparation was performed using the Nextera XT Library Preparation Kit (Illumina). Raw reads were processed with FastQC v0.74 (3) for quality assessment, and low-quality reads and adapters were removed using Trimmomatic v0.39 (4). After quality trimming, 5,213,764 high-quality reads were retained for genome assembly. *De novo* assembly was performed using Unicycler v0.4.8 (5), resulting in a draft genome comprising 86 contigs with an average sequencing depth of 106.50× coverage. The total genome length is 6,363,627 bp with a GC content of 66.46%, and the N50 value is 255,574 bp. The genome was annotated using the Prokaryotic Genome Annotation Pipeline v6.10 by NCBI (6), which showed the genome to contain 5,843 protein-coding genes, 3 rRNAs, and 58 tRNAs. Further analysis of the assembled genome using AMRFinderPlus 3.11.20 (7) and ResFinder v4.7.2 (8, 9) identified seven putative AMR genes in the bacterial isolate, namely, aph(3′)-Iib, blaOXA-396, blaPDC-67, blaPAO, catB7, tmexD2, and fosA. Two CRISPR arrays in the bacterial genome were revealed using CRISPR arrays and prophages by CRISPR immunity (7). PathogenFinder2 (10) and MLST v2.0 (11) explored it as a potential pathogen to humans with a score of 0.757 and belonging to sequence type 261, respectively. The metabolic functional features were identified by RAST (v.2.0) (12), where the assembled genome showed 392 subsystems with 29% coverage and 1,735 genes. Default parameters were used for all tools except where otherwise noted.

## Data Availability

The sequence reads are deposited in the Sequence Read Archive under accession number SRR36986717 and the assembled genome under accession number JBUCWI000000000. The associated BioProject and BioSample accession numbers are PRJNA1412003 and SAMN54809469, respectively. The output files from AMRFinderPlus, ResFinder, PathogenFinder, RAST, and MLST analyses have been made publicly accessible via Figshare at the following DOI: https://doi.org/10.6084/m9.figshare.31397613.
